# Collectanea

**Published:** 1829-01

**Authors:** 


### COLLECTANEA.

Floriferis ut apes in saltibus omnia libant,
Omnia nos, itidem, depascimur aurea dicta.

### PHYSIOLOGY.

*Central Point of the Nervous System.*—M. Flourens recently presented to the Academy of Sciences in Paris a Memoir entitled "Experiments on the Semicircular Canals of the Ear in Birds."

The author began by adverting to two Memoirs, not presented to the Academy, but published in the Annales des Sciences Naturelles, for January and February last. The object of the first is to determine with precision the limits of the central and vital point of the nervous system.

It results from his experiments that this point commences at the origin of the eighth pair of nerves, and extends over the space of a few lines only. By cutting the cerebellum below this point, its vitality ceases, yet the medulla spinalis is unaffected. Cut the spinal marrow below the point in question, and it dies. A point then exists in the nervous centres on which depends the life of all the other parts. This point is between the spinal marrow and cerebellum, the very centre of the nervous centres, (*au centre même des centres nerveux.*) It suffices that a part be united to this point to preserve its vitality: its death is the inevitable consequence of disunion.

*Reunion of the Ends of different Nerves.*—In the second Memoir, M. Flourens, after having repeated the experiments of Fontana, of Montana, of Cruickshank, and of others, on the reunion of the divided extremities of the *same* nerve, sought to determine the effects resulting from the union of the ends of different nerves. He therefore placed them in contact, and so kept them. In every instance the reunion took place. In some of the cases the return of the function was complete; in others it failed. In all, the transmission of irritations by the united nerves was perfect.

*Effects of the Section of the Semicircular Canal of the Ear in Birds.*—This is the immediate object of the Memoir now read.

The semicircular canals in birds are two vertical and one horizontal, which, with the vestibule and cochlea, form what is denominated the labyrinth, or internal ear.

In pigeons, the greater of these canals is the superior. It is vertical, and obliquely directed from behind forward. The middle is horizontal. The inferior is vertical, and directed from before; backward it crosses the horizontal.

M. Flourens, having successively made the section of these canals without producing the death of the animals, observed the following effects, which continued in many of them for nearly the space of twelve months.

1. The section of the *horizontal* canal of *both sides* is uniformly followed by a violent horizontal movement of the head The section of the vertical canal, whether superior or inferior, of both sides, is followed by a violent vertical movement of the head. Finally, the section of both the horizontal and vertical canals produced both the vertical and horizontal motion of the head.

2. The section of a canal on *one side* only, whatever be the canal cut, is accompanied by the motion of the head in a much smaller degree than when both sides are cut.

3. The section does not destroy life, but the effects above mentioned remain during the life of the animal.

4. The principle of this effect resides in the membranous lining and nervous expansion of the canals.

It is, says M. Flourens, an extraordinary fact that parts so small and of such delicate structure, should exercise so powerful an action on the animal economy; and it is equally so that parts, whose functions appear to be specially confined to the purposes of hearing, should have so marked an effect on the movements above described; and, finally, that each of the parts determines a motion in conformity with its own vertical or horizontal position. Thus, the horizontal section produces a horizontal motion; the section of the vertical is followed by the vertical motion.

### PATHOLOGY.

*Ulceration and Perforation of the Duodenum.* By M. Robert, Dresser at the Hôtel Dieu.—Louis Laurin, æt. seventeen, a shoemaker, and of a lymphatic constitution, had complained for several months of wandering pains at the epigastrium, accompanied six weeks with diarrhœa, and during the last ten days with anorexia, nausea, and general uneasiness. December 10th, 1827, after a meal rather more copious than ordinary, he suddenly felt an excruciating pain, which, commencing in the region of the stomach, soon extended over the whole abdomen, and caused him to vomit, first his food, and afterwards a sort of mucus covered with bile. A physician, who was immediately called in, thought the patient was affected with indigestion, and prescribed for him some camomile infusion, an anodyne enema, and cataplasms to the belly. During the night he remained in the same state, and had no alvine evacuations.

Next morning he was removed to the Hôtel Dieu. His countenance was pallid and altered, his skin covered with a cold sweat, his pulse frequent and small, his abdomen very painful and tense, his tongue pale and moist, and bilious vomiting occurred from time to time. Fourteen leeches were applied to the belly, and afterwards emollient fomentations, but with no benefit: for the patient died at four in the evening, after having suffered about twenty hours, dating from the invasion of the peritonitis.

On opening the body forty hours after death, the abdominal cavity was found distended with gases, and a serous, reddish, and very fetid fluid. The peritoneum, deprived of its natural appearance and suppleness, exhibited in divers points a red colour, disseminated in striæ or plates; and for the most part it adhered but slightly to the muscular coat of the intestines, from which it could be easily separated. In some places there was subperitoneal emphysema. The posterior cavities of the omenta contained no serous effusion, (*l'arrière cavité des épiploons;*) the intestinal circumvolutions were slightly agglutinated together. The mucous membrane of the stomach appeared sound. At the origin of the duodenum, immediately beyond the pylorus, there was an oval ulceration, three or four lines in diameter, with perpendicular edges, rather puckered, and of a greyish hue; the bottom of it was closed by the peritoneum, which had acquired a yellow tint, and presented a perforation a line in diameter, through which the peritoneal and intestinal cavities had free communication. Near this was discovered another ulceration, as large, but more superficial, since it did not penetrate beyond the mucous membrane. The rest of the alimentary canal was in a healthy state.—*Archives Generales de Medecine.*

---

*Suppuration of the Spleen.*—In the Observatore Medico for July, we find the following account:

Dominick Rotunno, ætat. twenty-nine, a miller of Genosa, near Tarentum, for some time had been affected with obstruction of the spleen subsequently to an intermittent fever. By excess of food and exercise, the tumor became larger and more painful, and ultimately was considered, by Dr. Gaetano Glionna, a well-marked case of splenitis. The disease acquired force, despite of venesection, and the abundant use of leeches, purgatives, and emetic tartar; the pain and tension of the hypochondrium increased, and were accompanied with rigors, succeeded by heat and nightly sweats, &c. The induration of the spleen now disappeared, and, augmenting in size, it became soft towards its inferior part, and assumed the general appearance of suppuration. Recourse was immediately had to emollient cataplasms; and the fluctuation was so evident in six days, that it became necessary to open the tumor directly. This was done by plunging a trocar into its centre, which was four inches from the linea alba. Nearly three pounds of very fetid pus flowed out instantaneously: it was rather firm, and at first of a dirty white, and afterwards of a reddish colour. The patient experienced immediate relief. The wound was kept open some days, but was quite healed in the course of a week; and Rotunno, entirely cured of his complaint, soon recovered his embonpoint and healthy appearance.—*Ibid.*

---

*Imperforation of the Vagina.* By Dr. CABARET, Basse Maison, near Glou-baray.—Marianne H., æt. twenty-one, of a robust make, had suffered, during five years, pains in the hypogastric region, with borborygmus and other hysteric symptoms, and had never had the menses. These various symptoms were successively combated by emmenagogues, antispasmodics, and the application of leeches to the superior and inner part of the thighs; but none of these means produced any salutary change.

It was then that the patient determined to consult Dr. Cabaret, and she accordingly did so in the beginning of April, 1825. On feeling the hypogastrium with attention, he fancied he could distinguish a very voluminous tumor in the situation of the uterus, which at first induced him to believe that the young woman was really pregnant, and attempting to deceive him; but her good character, and the assurance which she gave of never having menstruated, soon led him to adopt a different opinion. On minutely examining the parts of generation, he discovered a thick membrane, by which the vaginal orifice was completely closed. The incision of this membrane was evidently the indication to be fulfilled, which operation Dr. C. executed thus: Having placed the patient in a convenient posture, he separated the labia with the index and middle fingers of the left hand, and with a bistoury in the right, he made a crucial incision. Four pounds of blackish blood, mixed with some extremely fetid coagula, immediately issued forth. Some lint was introduced between the divided edges, to prevent their adhesion; and the next day injections were thrown into the vagina, for the purpose of dissolving and bringing away any coagula of blood which might still remain there, and fresh lint was introduced, and kept in situ by a suitable bandage.

The hypogastric tumor had entirely disappeared, and likewise all the symptoms which it occasioned.

By continuing the same dressing for a fortnight, Marianne H. was thoroughly cured, and she has ever since enjoyed healthy and regular menstruation.—*Ann. de la Med. Phys.*

---

*Lachrymal Calculus.* By M. KRIMER.—A woman, in her thirty-second year, having been for nine months affected with fistula lachrymalis, applied to M. Krimer. By introducing a probe into the fistula, he discovered that the lachrymal sac was not ulcerated, but the nasal duct closed by a hard substance, which he imagined to be an exudation of o seous matter. He vainly attempted to remove the obstruction by forcing a passage with a pointed probe. Upon endeavouring, however, to withdraw the instrument, he met with a resistance for which he was at a loss to account; and, when he had at length extricated the probe, he was not a little surprised to find a calculus, of the size of a pea, adhering to its point. The nasal canal, being again explored, was found quite pervious, and was for a fortnight kept free by the introduction of a bougie. Afterwards the wound was allowed to cicatrise.—*Graefe and Walther's Journal.*

### PRACTICAL MEDICINE.

*The Efficacy of Liquor Ammonia in Cases of Intoxication.*—A mendicant, between twenty and thirty years old, and nearly in a state of idiocy, entered a public-house where several young men were assembled to drink. They diverted themselves by supplying him with wine and brandy till thoroughly intoxicated, and, scarcely able to stagger a few paces, he fell down apparently dead. He remained two hours in an alarming state of insensibility, in despite of various means employed to rouse him. His case seemed to offer a favorable opportunity for ascertaining the merits of the volatile alkali. Eight drops of the Liquor Ammonia were given to him in a glass of cold water, but of this a quarter was spilt, owing to his difficulty of swallowing. From the moment it was given to the patient he appeared somewhat convulsed; shortly afterwards, he was observed to stretch himself; before five minutes had elapsed, he sat upright, and was entirely recovered from the lethargy in which he had been absorbed. His eyes were fixed, and resembled those of a person suddenly awaked from a dream. He sighed several times, shed some tears, stammered out an excuse, and was in a few minutes strong enough to return home, leaning upon the arm of one of the bystanders. In the course of an hour he was quite restored, with the exception of a slight degree of torpor which still remained. It was thought proper to try the effects of a second dose of ammonia, and four more drops reestablished him.—*Journal de la Soc. Royale de Med. Chir. et Pharm. de Toulouse.*

---

*Symptoms of a Convulsive Epidemic similar to that which now infests the City of Paris.*—The winter of 1769-70 was prolonged far into the spring, and the previous summer was noticed for the variety of its temperature and its fluctuations of heat, cold, and moisture. The disease began in the month of September, but its severity was more manifest in the spring. In some the attack was sudden and rapidly fatal, which differs from the present epidemy. The second was of a more chronic species, was also more violent than that of the present period, but possessed many points of resemblance to it.

The disease in this form was preceded by weight and numbness in the arms and legs. The patients were depressed in spirits, and experienced uneasiness about the stomach; about the same time, a sense of formication all over the limbs. Unless the symptoms were arrested by suitable treatment, they continued to increase. The colour of the skin was yellow, or earthy. The appetite was invariably good, sometimes voracious at the end of the disorder. Spasms took place in various parts of the body, principally in the forenoon. Rigidity and numbness of the fingers were among the symptoms, and, as long as this continued, whatever abatement might be experienced in other respects, the patient carried about him the essence of the disease; or, to use the words of the analyst of the original work, "le feu couvait encore sous la cendre."

The patients, even in their tranquil moments, had dilated pupils and weakness of vision, and at the same periods tremblings of the arms were remarked, in those especially who had been frequently bled.

When the spasms had frequently recurred, the ends of the fingers and toes became so insensible that the patients could handle lighted coals without feeling the heat.

The disease never terminated by gangrene, but the epidermis disquamated in different parts.

*Treatment.*—After emetics and purgatives, among which calomel appeared to have the best effect, TAUBE had recourse to calmants: the most efficacious was a mixture of six gros of camphor, a pint of good vinegar, and three ounces of extract of Genièvre; two tablespoonfuls were given every two hours.

Decided benefit was experienced from the use of Hensler's powder, composed of equal parts of Calamus Aromaticus, Galanga, Pied de Veau, and wild Valerian, with a small portion of rhubarb and of camphor.

One patient was snatched from the jaws of death by musk.

Opium procured only temporary relief. Leeches were useful in dissipating pain in the limbs which were affected by spasm.

Frictions with essence of turpentine, and electric shocks, were successfully employed to remove the numbness and insensibility of the feet and hands.—*Die Geschictete des Kriebel krankheit, &c.* Gottingen, 1782. See also *Commentarii de Rebus in Scientiâ Naturale et Medecina gestes*, t. 25. p. 532.

In 1588, 1593, and 1596, a similar disease reigned in Silesia, and is thus described in a treatise entitled "De Morbo Convulsivo, Maligno, et Epidemico," inserted by GREGOIRE HORST among his "Observations," tome ii. p. 299.

The patients experienced at the commencement a pricking in various parts of the body, as if thousands of ants were traversing them. Cramps were felt, accompanied by intolerable pains. The symptoms sometimes arose spontaneously, at others were preceded by vomitings of viscid matter, which nevertheless was unaccompanied by pain in the stomach, or any other gastric symptom. The affection was sometimes confined to the limbs for several days, and even months; but in some cases it extended to the head, producing convulsions and epilepsy. The spasms sometimes affected the respiratory organs, so as to cause apparent death. The prolongation of the malady brought on tumefaction of the feet, with phlyctenæ, from which a copious discharge of ichor was produced.

HOFFMAN notices similar epidemics; and our countryman WILLIS describes one in his treatise on Morbis Convulsivis. In 1702, it overran the whole country of Frieberg. In 1716, it desolated Saxony and Lusace, spread itself into different parts of Germany, Silesia, Wirtemberg, and Bohemia.

MULLER gives a good description of the same disease in his "Disputat. de Morb. Epidem."

### SURGERY.

*Remarkable Superiority of Amputating by one Incision through the Integuments and Muscles.*—This great improvement in the mode of amputating, which was formerly recommended by LOUIS, and unaccountably abandoned, is practised and highly recommended by all the present surgeons of the Hôtel Dieu, M. DUPUYTREN, M. BRESCHET, and M. SANSON. In thighs removed by the two former, and an arm by the latter, no tourniquet was employed in either case. The limb was grasped by an assistant, and pressure made on the principal artery. A single incision cut through skin and muscles down to the bone, and a retraction of the skin and muscles not inserted in the bone was effected to the extent of three inches. The first retraction having been completed, the muscles attached to the bone were cut through by a scalpel, on a level with the others, and the bone sawed as usual. The stumps in all were remarkably fine, and the extremity of the bone was more than sufficiently covered.

After each operation, the surgeons respectively explained their reasons for preferring this procedure, whose expediency was proved by the result of every case in which it has been tried of late. To say nothing of the discarding of the tourniquet, which enables the operator to effect a retraction of the muscles to a greater extent than under its use; the old method of preserving the skin by cutting it up from the muscles to which it naturally adheres, was a considerable impediment to union by the first intention.

---

*Erysipelas Phlegmonodes treated by Compression.* By M. Bougon.—This practice, which originated with some of the most eminent of the French surgeons, continues to be employed with success at the Hospice de Perfectionnement.

A woman, sixty-five years of age, was received with a leg swoln, painful, and of a red colour verging upon brown. The pain was increased on pressure; the redness was not diminished by it; and the subcutaneous cellular texture felt like dough. The knee was also swoln, and the synovial membrane appeared to contain liquid. The inflammation soon extended to the thigh. The decrepitude of the old woman contraindicated the use of evacuants; M. Bougon, therefore, ordered compression of the entire limb to be methodically made, and renewed whenever the bandages might become loose. The pressure at first produced pain, which was speedily dissipated; and this extensive phlegmonous erysipelas, which took place in a subject most favorably disposed for its passing into a gangrenous state, happily terminated by resolution.

Case II.—An old man, sixty years of age, had both legs of an extraordinary bulk; the skin was tense and painful, and of a brownish red colour, which did not disappear on pressure. Two bleedings were performed on account of supposed tendency to apoplexy, but they had no effect on the erysipelas. Methodical compression of the limbs by bandage was had recourse to, whereby they were cured in six days.

